# Optimizing solvent systems for electrospun PLGA scaffolds: effects on microstructure and mechanical properties for biomedical applications

**DOI:** 10.1039/d4ra07881k

**Published:** 2025-01-31

**Authors:** Golestan Salimbeigi, Garrett B. McGuinness

**Affiliations:** a School of Mechanical and Manufacturing Engineering, Dublin City University Dublin 9 Ireland garrett.mcguinness@dcu.ie

## Abstract

Electrospun scaffolds fabricated from poly(lactic-*co*-glycolic acid) (PLGA) have garnered widespread interest in biomedical applications due to their ability to mimic the extracellular matrix (ECM) structure with a tunable degradability profile. The properties of electrospun scaffolds are meticulously tailored for specific applications through the adjustment of polymer properties, solution parameters, and processing conditions. Solvent selection is crucial, influencing polymer spinnability and scaffold topographical, physical and mechanical features. Hansen solubility theory aids in predicting suitable solvent systems. The absence of specific data prompted a solubility experiment to determine Hansen solubility parameters for PLGA. Subsequently, various solvent systems were investigated for their impact on the microstructure of electrospun PLGA scaffolds. Optimizing the electrospinning process resulted in fibrous scaffolds with consistent average fibre diameter from different solvent systems, allowing a focused examination of the solvent's isolated influence on mechanical properties. PLGA samples electrospun using hexafluoro isopropanol (HFIP) displayed lower Young's modulus and ultimate tensile strength but higher failure strains than those created using binary solvent systems composed of tetrahydrofuran (THF), dichloromethane (DCM), and dimethylformamide (DMF). This research advances the understanding and optimization of electrospun PLGA scaffolds, enhancing their potential for biomedical applications.

## Abstract 2

Optimizing solvent systems for electrospun PLGA tissue scaffolds enables control over mechanical properties. Hansen solubility theory helps identify key solvent effects, enabling the design of tunable, high-performance scaffolds for biomedical use.

## Graphical abstract

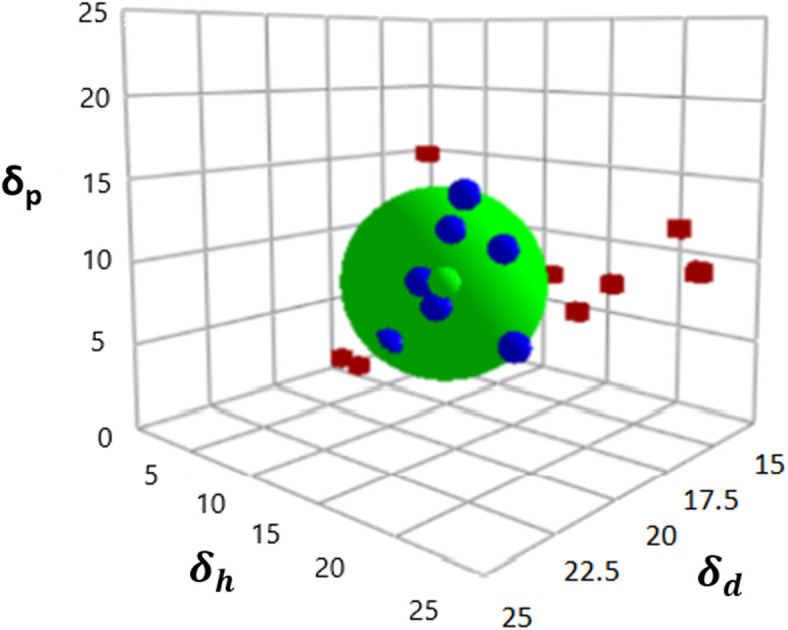

## 1. Introduction

Electrospinning, a technique for crafting fibrous scaffolds, has captured the interest of researchers for its adeptness in faithfully reproducing native ECM structures.^1–3^ This technique produces continuous fibres with diameters ranging from microns to nanometres, closely mirroring ECM topography, which significantly influences cell functions and biomechanical forces.^4–6^ The relevance of this technique extends to various biomedical applications, including wound healing, drug delivery, and tissue regeneration and regenerative medicine, where the scaffold's ability to mimic physical, mechanical, and topographic features of the native ECM plays a crucial role in promoting effective cellular responses.^7–10^ Electrospinning, as a process for generating nanofibres from polymer solutions, employs a robust electric field to form a fine jet that undergoes stretching and whipping motions before solidifying into nanofibres.^11,12^ This approach enables the creation of nanofibres with adjustable physical and mechanical properties, rendering it valuable in biomedical applications including tissue engineering.^13–15^

The literature on electrospinning focuses heavily on the processing of both natural and synthetic polymers. While natural ECM proteins like collagen and gelatin have desirable biological properties, their low molecular weight, poor solubility, and mechanical limitations hinder their use in scaffold production.^16–18^ Previous studies have highlighted the benefits of blending these natural polymers with synthetic biocompatible polymers, which not only enhance mechanical properties but also support cellular interactions. Researchers have extensively investigated blending these natural polymers with biocompatible polymers including polylactide (PLA), PLGA, and polycaprolactone (PCL).^18–20^ Among these, PLGA has emerged as a highly versatile material due to its adjustable degradation rate, mechanical properties making it suitable for a variety of biomedical applications.^21–23^ This versatility allows for tailored scaffolding solutions that can be customized to meet specific requirements, thus broadening the scope of their application. This tailoring is achieved through the careful adjustment of parameters such as polymer composition, solution characteristics, and processing conditions, enabling precise control over the scaffold's structure and function to meet diverse biomedical needs.^24–26^ Electrospinning heavily relies on the solvent system, influencing not only the polymer's spinnability but also the microstructure and mechanical properties of the resulting fibres.^27–29^ However, a significant gap in the literature concerns the effect of solvent systems on the electrospinning process regarding the aforementioned properties, particularly for PLGA. Studies have shown that different solvent systems can yield diverse fibre morphologies and microstructures, yet comprehensive investigations on linking these changes to specific performance outcomes remain sparse. While some studies have explored solvent effects on fibre morphology, few have provided comprehensive investigations of how these effects translate into mechanical properties.

Moreover, the strong intermolecular bonds in PLGA present unique challenges in finding suitable solvent systems for homogeneous solution formation, particularly in complex mixtures involving multiple solvents. Addressing this gap is crucial for optimizing the performance of electrospun scaffolds in biomedical applications, as the choice of solvent can significantly affect both the microstructure and the functionality of the final product.

Despite advancements in electrospinning, a thorough investigation into the specific interactions between solvent systems and PLGA microstructure remains unrealised. Key unresolved issues include identifying solvent systems that consistently produce scaffolds with the desired mechanical properties, such as tensile strength and modulus, while maintaining a uniform fibre diameter. This exploration is essential not only for improving the reproducibility of scaffold fabrication but also for ensuring that the resultant structures can effectively mimic the ECM. Addressing these gaps is critical because scaffold performance is not solely dependent on fibre diameter but also on the microstructural and mechanical consistency of the fibres.

Our research builds upon these prior studies by applying Hansen solubility theory, which allows for a more accurate prediction of solvent compatibility based on dispersion, polar, and hydrogen bonding components.

Solubility, governed by chemical structure, involves thermodynamic considerations, with Gibbs free energy playing a key role.^30^ Hildebrand's theory correlates the internal pressure of solvents with the solubility order, emphasizing the importance of cohesive energy density (CED) and Hildebrand solubility parameters.^31^

The Flory–Huggins theory introduces the polymer–solvent interaction parameter *χ*, which comprises enthalpic (XH) and entropic (XS) components. Hildebrand parameters are linked to XH, and the theory is applicable to non-polar systems. However, it has limitations, especially regarding polar intramolecular bonds.^32–34^

The Hansen three-dimensional solubility parameters (HSP) provide a more comprehensive approach, considering dispersion (*δ*_d_), polar (*δ*_p_), and hydrogen bonding (*δ*_h_) components. HSP for polymers are determined indirectly by testing materials with various solvents. This involves a three-component graphing system, often visualized as a sphere, with coordinates representing *δ*_d_, *δ*_p_, and *δ*_h_ values of the polymer. The solubility parameter distance, *R*_a_, is crucial and can be calculated using the equation:

1 *R*_a_^2^ = 4(*δ*_d,p_ − *δ*_d,s_)^2^ + (*δ*_p,p_ − *δ*_p,s_)^2^ + (*δ*_h,p_ − *δ*_h,s_)^2^

Solvents within the solubility sphere (*R*_a_ < *R*_0_) are likely to dissolve the solute, while poor solvents lie outside the sphere.^35^

A two-dimensional (2D) graph, such as Bagley's graph, represents Hansen's 3D volume without omitting any component.^36^ Bagley's model introduces a new component, *δ*_v_, given by:2
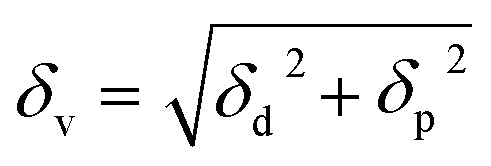


This model presents a “solubility sphere” of a polymer as a circle in a 2D system, with axes represented by *δ*_h_ and *δ*_v_.^36^

In this study, we first established HSP for PLGA, followed by exploring multiple solvent systems to assess their effects on the microstructure of electrospun scaffolds. Through meticulous optimization of the electrospinning process, consistent fibre diameters were achieved across different solvents, enabling a detailed investigation into how solvent choice influences mechanical properties. This investigation unveils how solvent selection directly influences the microstructure of electrospun PLGA scaffolds, demonstrating its independent impact on mechanical properties beyond fibre diameter. These insights promise refined strategies for optimizing scaffold performance tailored to biomedical applications.

## 2. Experimental

### 2.1 Materials

PLGA (50-50), *M*_w_ of 65 000 Da, was purchased from Ashland Specialties Ireland Ltd. Solvents used within this study were obtained from Sigma Aldrich, USA the list of which and their properties are detailed in Table 1. All reagents were of analytical grade and were used as received.

**Table 1:** Physical properties of the solvents^a^^37–40^

Solvent	Boiling temperature (°C)	Electrical conductivity at 25 °C (S m^−1^)	Surface tension at 20 °C (mN m^−1^)	Dielectric constant at 20 °C	Viscosity at 25 °C (cP)	Vapour pressure at 25 °C (kP)
HFIP	59	—	15	15.7	1.65	16
CHL	58	<1.0 × 10^−8^	27.14	4.8	0.54	25
THF	66	4.5 × 10^−5^	28	7.6	0.36	19
DMF	153	6.0 × 10^−8^	36.76	36.7	0.92	0.516
DCM	40	4.3 × 10^−11^	28.12	9.1	0.43	53
TFA	72.4	—	72.5	42.1	1.8	1.17
Acetone	56.05	0.5 × 10^−6^	23.7	25	0.29	30
Ethanol	78	1.4 × 10^−9^	22.3	22.4	1.2	5.95
Methanol	64	1.5 × 10^−7^	22.6	32.6	0.545	13
Acetic acid	118	6 × 10^−7^	27.4	6.2	1.13	2.1

a HFIP, CHL, THF, DMF, DCM, TFA denote hexafluoro isopropanol, chloroform, tetrahydrofuran, dimethylformamide, and trifluoroacetic acid, respectively.

### 2.2 Establishing Hansen solubility parameters for PLGA and creating a solubility graph

This experiment involved using various solvents with different HSP to determine the HSP for PLGA (see Table 3). One millilitre of each solvent system was pipetted to 0.1 grams of the polymer. The vials were left for 24 hours without stirring and then were labelled 0 for undissolved and 1 for a clear solution. The HSPiP 5th edition 5.3.04 software was used for calculating the HSPs by best fitting the data to a spherical curve the centre of which gives the Hansen solubility parameters for PLGA. Utilizing the generated HSPs for PLGA, a Bagley's graph specific to PLGA was constructed, aiding in the identification of the most suitable solvents for the electrospinning process.

### 2.3 Preparation of electrospinning solutions

The electrospinning solutions were prepared by first dissolving PLGA in the specified solvent system. The quantities of polymer were calculated based on a w/w system in line with the target concentrations detailed in Table 2. After stirring for 2 hours at room temperature (21 ± 2 °C), surfactants were added to certain solutions based on the solvent-to-surfactant volume/volume (v/v) ratios provided in Table 2. The solutions were then stirred for an additional 22 hours to achieve complete mixing and homogeneity.

**Table 2:** PLGA solution parameters (concentration and surfactant screening trails)^a^

Abbreviation	Concentration (w/w%)	Solvent system	Solvent/surfactant ratio (v/v)
PG-TD8 (7-3)	8	THF-DMF	7-3
PG-TD10 (7-3)	10	THF-DMF	7-3
PG-TD12 (7-3)	12	THF-DMF	7-3
PG-TD14 (7-3)	14	THF-DMF	7-3
PG-TD14 (1-1)	14	THF-DCM	1-1
PG-TD16 (1-1)	16	THF-DCM	1-1
PG-DD10 (7-3)	10	DCM-DMF	7-3
PG-DD10 (1-1)	10	DCM-DMF	1-1
PG-DD12 (1-1)	12	DCM-DMF	1-1
PG-HFIP6	6	HFIP	—
PG-HFIP8	8	HFIP	—
PG-HFIP10	10	HFIP	—
PG-HFIP12	12	HFIP	—

a PG, TD, DD, denote PLGA, THF-DMF, DCM-DMF, respectively.

### 2.4 Electrospinning

The prepared PLGA solutions were added to a 20 ml glass syringe with 20 G attached stainless steel blunted needle. 100 mm square aluminium plates with a thickness of 0.25 mm (Goodfellow) were used as the collector targets. A positive bias was applied to the solution *via* a voltage source and an electrical ground connection was attached to the rear surface of the plate. A high voltage probe (Radionics, Ireland) was used to ensure a correct voltage bias was achieved. A syringe pump (KDS, US) was used to dispense the polymer solution at a controlled feed rate from the capillary. All electrospinning experiments were conducted within a fume hood cabinet to extract evaporating chemicals. The electrospinning process was conducted at a voltage of 15 kV, a flow rate of 0.25 ml h^−1^, and a distance of 15 cm, constant relative humidity (60–65%), and at room temperature.

### 2.5 Measurements and characterization

#### 2.5.1 Surface morphology

The surface morphology of the electrospun fibres was examined by scanning electron microscopy (EVO LS15 SEM). Scaffolds were sputter-coated with gold prior to imaging and were typically viewed with an acceleration voltage ranging from 10 to 15 kV. The average diameter and pore size of the fibres were manually measured from the SEM images using Image J software (National Institute of Health, USA) for 100 randomly selected fibres from 4 samples.

#### 2.5.2 Porosity analysis

The porosity of the electrospun meshes, with sample dimensions of 10 mm × 10 mm and a thickness of 15 ± 3.2 μm, was calculated using the following equation:3
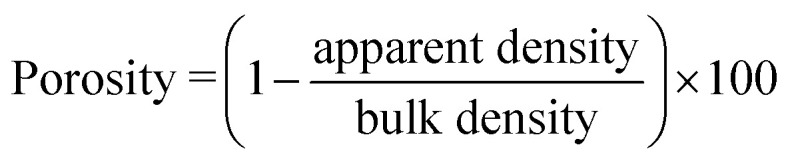


The bulk density of PLGA was taken as 0.615 g cm^−3^. The apparent density of the specimens (*n* = 4) was determined using a density determination kit with a measuring accuracy of ±0.1 mg (0.0001 g) based on the buoyancy technique. The technique works based on the Archimedean principle to measure the density of the scaffolds and can be calculated from eqn (4)4
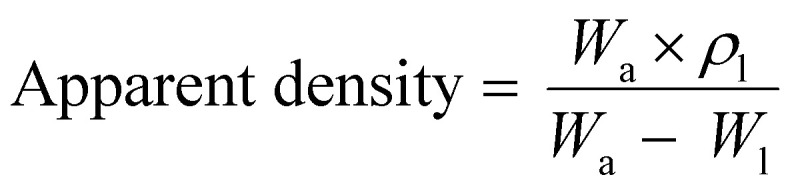
where *ρ*_l_ is the density of the liquid (distilled water in this experiment), *W*_a_ is the weight of the solid in the air, and *W*_l_ is the weight of the solid in the liquid. The temperature of the distilled water was measured using a thermometer and the density of water was corrected accordingly to avoid any error.

#### 2.5.3 X-ray diffraction

X-ray diffraction was performed on PLGA electrospun scaffolds using a Brüker D8 Advance X-ray diffractometer to determine the crystallinity of the fibrous structures. The scaffolds were secured on the machine sample holder using a vacuum system. A blank scan of the holder was made before measurement to provide a baseline scan. Scanning was performed over a 2*θ* range of 10 to 60°, in 0.05° increments at a scan rate of 1° per minute.

#### 2.5.4 Biodegradation

Scaffolds were prepared for biodegradation analysis by punching ø2 cm discs from the collected fibre scaffolds using a custom-built punch. Each specimen was placed in a container that contained 30 ml of 0.1 M phosphate-buffered saline (PBS, pH 7.4). The specimens were stored in an incubator (37 °C) for desired periods. The PBS solution was replaced with fresh solutions weekly. The degradation test was carried out for four weeks. Harvested specimens were washed with distilled water three times and left to be air-dried for characterisation.

#### 2.5.5 Mechanical properties

Uniaxial tensile testing of ‘dogbone’ specimens was performed on selected fibrous scaffolds. Samples were prepared using a custom-designed and manufactured stainless steel cutting die. Briefly, the die consisted of a sample gauge length of 16 mm and a test width of 3.9 mm. Samples were punched from the spun scaffolds (16 mm × 3.9 mm) using the cutting die and a custom-built sample holder. Tensile testing was conducted using a Zwick Z005 tensile test machine with a 500 N load cell attached. The thickness of the test sample was measured using a micrometre (Draper Tools, UK) before being secured within the test grips, with the thickness of 15 ± 3.2 μm. A small piece of sandpaper was placed on either side of the grip areas to ensure no slipping of the specimen occurred during testing. The testing cycle consisted of an initial preload of 0.05 N to remove any slack from the sample, with the grip separation at this point used as the gauge length. Following this, the sample was elongated at a strain rate of 2 mm min^−1^ with failure determined to occur when the force dropped to 80% of the max force value recorded.

## 3. Statistical analysis

All quantitative results were obtained from at least three samples for analysis. Data were expressed as the mean ± standard deviation. The differences were compared by student's *t*-test. A value of *p* < 0.05 was considered to be statistically significant.

## 4. Results and discussion

### 4.1. Establishing HSP and solubility graph for PLGA

**Fig. 1:**
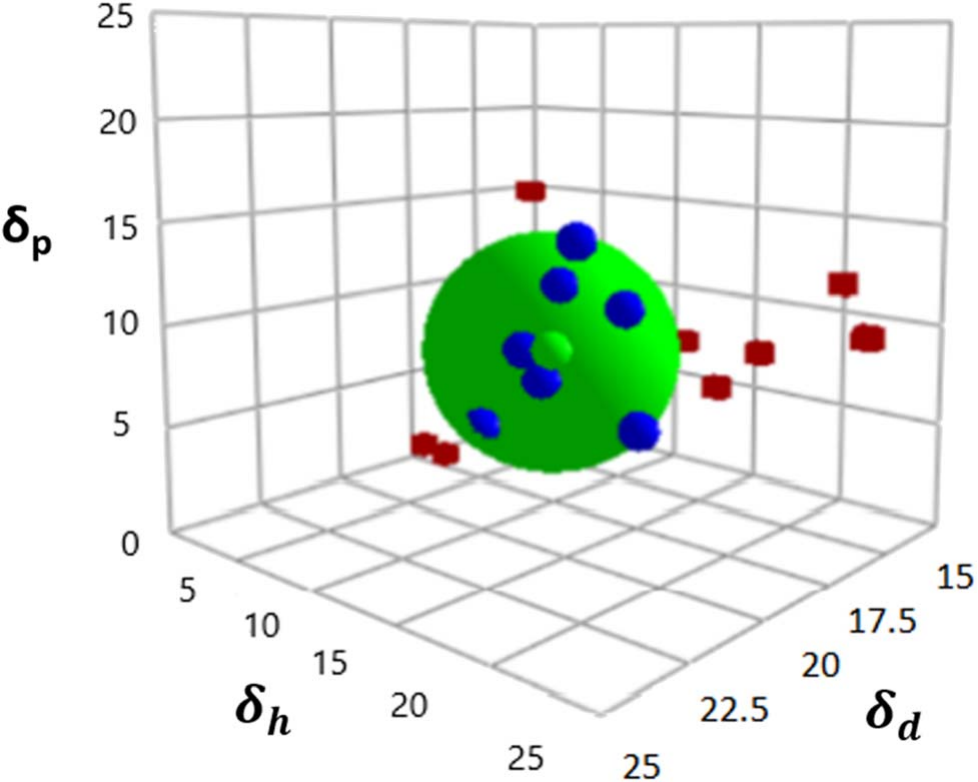
Hansen solubility graph for PLGA using HSPiP 5th edition 5.3.04 package, *δ*_d_, *δ*_h_, *δ*_p_ denote dispersion, polar, and hydrogen components, respectively.

Fig. 1 displays the Hansen solubility graph for PLGA, created by optimally fitting the data points shown in Table 3. In this graph, the red cubes represent solvents with a solubility state labelled as 0, indicating poor solubility, while the blue spheres represent solvents with a solubility state labelled as 1, indicating good solubility.

**Table 3:** Results of dissolution experiments for all combinations of polymer and solvent

Solvent system	Solubility status
HFIP	1
DCM	1
CHL	1
THF	1
Acetone	1
TFA	1
DMF	1
Ethanol	0
Acetic acid	0
Propanol	0
CHL/ethanol (1-1)	0
DCM/acetone (1-1)	1
HFIP/DMF (1-1)	1
HFIP/acetic acid (1-1)	1
DCM/ethanol (1-1)	0

The coordinates of the centre of the developed solubility sphere and its radius provide the HSP (*δ*_D_, *δ*_P_, *δ*_H_) and the interaction radius for PLGA. Specifically, the HSP for dispersion forces (*δ*_D_), polar forces (*δ*_P_), and hydrogen bonding forces (*δ*_H_) are 17.6, 7.8, and 9.62, respectively, with an interaction radius of 6.2.

This graph visually depicts the solubility space of PLGA, illustrating which solvents can dissolve PLGA effectively (blue spheres within the sphere) and which cannot (red cubes outside the sphere).

Table 4 presents the Hansen solubility parameters and the determined *R*_a_ values of the selected solvents for dissolving PLGA, calculated using eqn (1). The solvents are ranked by their dissolving power for PLGA as follows:THF > DCM > TFA > acetone > HFIP > CHL > DMF > ethanol > methanol

**Table 4:** Comparison of Hansen solubility parameters of the solvents for dissolving PLGA, *δ*_d_, *δ*_p_, *δ*_h_, *δ*_t_ denote Hansen dispersion, polar, hydrogen and total solubility parameters, *δ*_v_, *R*_a_ are Bagley's component and solubility parameter distance

	PLGA	HFIP	CHL	THF	DMF	DCM	Ethanol	Methanol	Acetone	TFA
*δ*_d_ (MPa^1/2^)	17.6	17.2	17.8	16.8	17.4	18.2	15.8	14.7	15.5	15.6
*δ*_p_ (MPa^1/2^)	7.82	4.5	3.1	5.7	13.7	6.3	8.8	12.3	10.4	9.7
*δ*_h_ (MPa^1/2^)	9.62	14.7	5.7	8	11.3	6.1	19.4	22.3	7	11.4
*δ*_v_ (MPa^1/2^)	19.25	17.7	20.3	17.74	21.9	19.259	18.08	19.16	18.66	18.36
*δ*_t_ (MPa^1/2^)	21.52	26.68	18.94	19.46	24.7	20.2	21.9	29.44	19.93	21.61
*R*_a_	6.2	6.12	6.14	3.1	6.32	4.01	10.4	14.64	5.58	4.764
Solvent power		Good	Good	Very good	Good	Very good	Poor	Poor	Good	Good

To provide an easier and more intuitive visualization of solvent compatibility, a Bagley diagram is employed. This two-dimensional representation simplifies the interpretation of solubility data by projecting the three-dimensional solubility sphere onto a plane, allowing for a clearer assessment of solvent effectiveness.

**Fig. 2:**
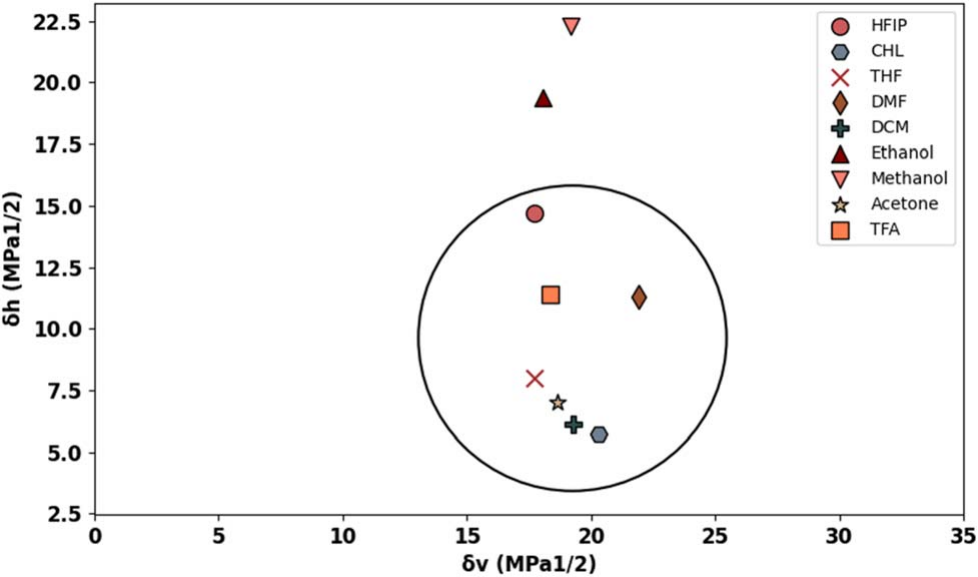
Bageley solubility graph of PLGA.

The Bagley diagram of PLGA, shown in Fig. 2, graphically represents the solubility sphere with a radius of 6.2. This diagram illustrates that, apart from methanol and ethanol, whose points lie outside the solubility sphere, all other investigated solvents are compatible with PLGA. This compatibility is also reflected in the calculated solubility radius, demonstrating the effectiveness of these solvents in dissolving PLGA (see Table 4).

Among the investigated solvents, THF and DCM emerge as the optimal choices for dissolving PLGA. However, when aiming for tunable and defect-free fibres using electrospinning, additional solution criteria such as conductivity, surface tension, and vapor pressure must be considered. For instance, DCM, with a high vapor pressure of 53 kPa, poses challenges for the electrospinning process due to its low dielectric constant of 8.93, necessitating improvements in the solution's electrical conductivity. Similarly, THF has a low dielectric constant of 7.6, which also requires attention.

To address these issues, the incorporation of surfactants or a second solvent becomes necessary. DMF displays favourable solubility compatibility with PLGA, good electrical conductivity, and low vapor pressure, making it a suitable choice as the second solvent in binary solvent systems. HFIP was also selected as the third solvent, given its compatibility with PLGA and suitable vapor pressure, dielectric constant, and surface tension. This comprehensive analysis underscores the importance of considering multiple solvent properties beyond solubility alone when developing polymer solutions for electrospinning applications.

### 4.2 Effect of solvent type on the morphology of PLGA nanofibres

**Fig. 3:**
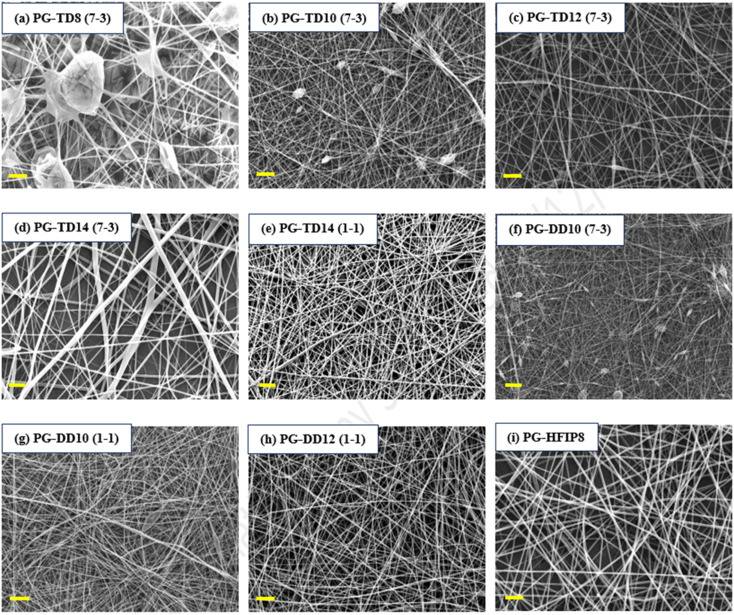
SEM images of the electrospun PLGA fibres produced from different solvent systems including (a) 8 w/w% THF-DMF (7-3), (b) 10 w/w% THF-DMF (7-3), (c) 12 w/w% THF-DMF (7-3), (d) 14 w/w% THF-DMF (7-3), (e) 14 w/w% THF-DMF (1-1), (f) 10 w/w% DCM-DMF (7-3), (g) 10 w/w% DCM-DMF (1-1), (h) 12 w/w% DCM-DMF (1-1), (i) 10 w/w% HFIP; scale bar: 10 μm.

To examine how different solvent systems, affect fibre morphology, PLGA was dissolved in THF-DMF, DCM-DMF, and HFIP. Fig. 3 shows SEM images of the resulting electrospun fibres that were prepared from these solvent blends. In the case of 8 w/w% PLGA in THF-DMF (7-3, v/v), large beads were visible within the nanofibre structure due to insufficient polymer chain entanglement at this concentration. Increasing the concentration to 10 w/v% improved fibre morphology, though small beads remained on the fibre surface. A concentration of 12 w/w% yielded higher-quality fibres, albeit with some small spindle-like beads present. Further increasing the concentration to 14 w/w% resulted in smooth, bead-free fibres, though with an increase in fibre diameter and decreased packing density. To address this, the surfactant quantity was increased to 50% (THF-DMF: 1-1, v/v) while maintaining the solution concentration at 14 w/w%, resulting in highly uniform and thinner fibres compared to the initial 14 w/w% solution.

For the DCM solvent system, 10 w/w% PLGA in DCM-DMF (7-3, v/v) produced highly beaded fibres, indicating a need for improved electrical conductivity. Increasing the amount of DMF in the solution (DCM-DMF, 1-1, v/v) at 10 w/w% concentration significantly improved fibre morphology and reduced bead formation. However, some beads were still present on the fibres, prompting an increase in solution concentration to 12 w/w%, resulting in smooth, defect-free fibres (PG-DD12, 1-1, v/v).

Conversely, HFIP facilitated the production of quality fibres at lower concentrations compared to THF and DCM. The most uniform and high-quality fibres were obtained from this solvent system (PG-HFIP8).

It's worth noting that for comparative analysis, solution concentrations are typically kept similar to attain solutions with comparable viscosity. However, viscosity is influenced by factors such as solvent nature. THF and DCM possess lower viscosities (0.36 and 0.43 cP, respectively) compared to HFIP (1.65 cP). Therefore, higher PLGA concentrations were required in DCM and THF solvents compared to others.

### 4.3 Impact of solution concentration on the microstructure of PLGA scaffolds using HFIP

**Fig. 4:**
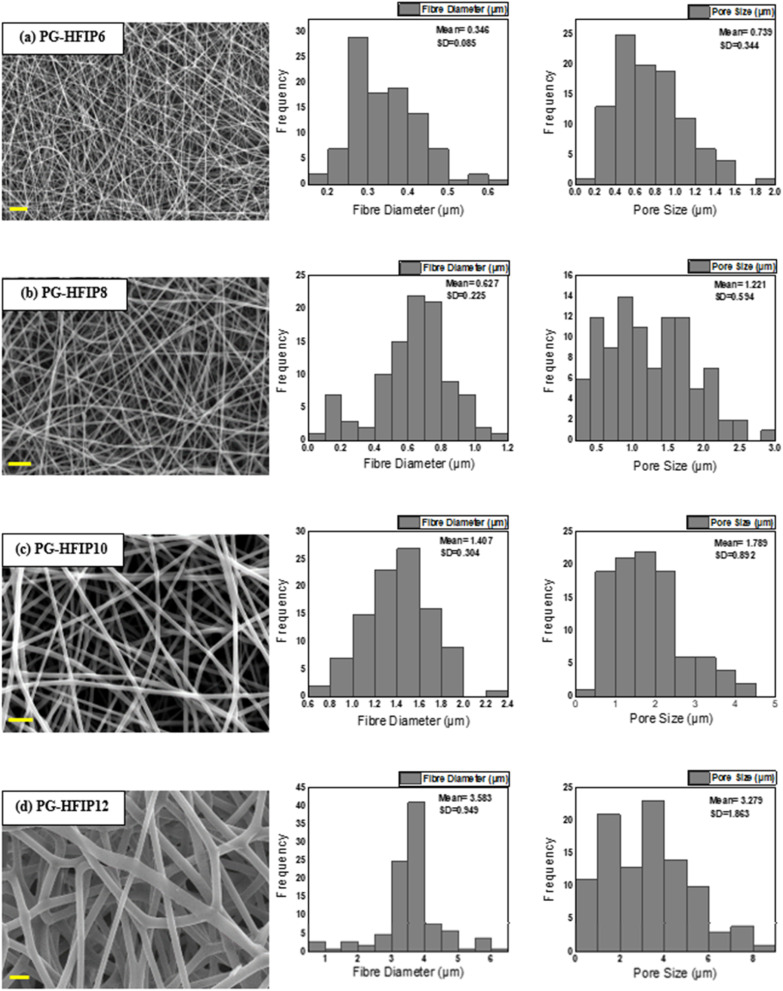
SEM images and corresponding fibre diameter and pore size distribution graphs of PLGA scaffolds produced from differently concentrated solutions from HFIP including (a) 6 w/w%, (b) 8 w/w%, (c) 10 w/w%, (d) 12 w/w%; scale bar: 10 μm.

**Fig. 5:**
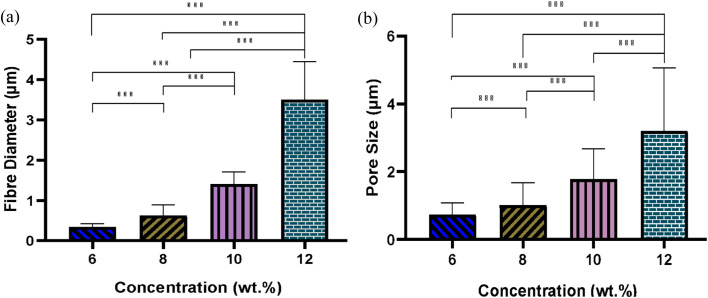
Effect of solution concentration on (a) average fibre diameter and (b) pore size of PLGA fibres from HFIP solvent system; (***P < 0.001).

Fig. 4 illustrates the morphology, corresponding fibre diameter, and pore size of electrospun fibres from PLGA/HFIP solutions at varying concentrations. Fibres produced from this solvent system exhibit flawless, smooth, and uniform characteristics across all concentrations. Analysis of distribution graphs reveals an increase in fibre diameter from 0.346 ± 0.085 μm for the 6 w/w% solution to 3.583 ± 0.0949 μm for the 12 w/w% solution. Inter-fibre pores within electrospun scaffolds represent the spaces between the deposited fibres, which are expected to enlarge as the fibre diameter increases. In the context of tissue engineering applications, these inter-fibre pores are critical for cellular infiltration, as they provide the necessary pathways for cells to migrate and populate the scaffold. Therefore, optimizing the size of these pores is important for ensuring the scaffold can effectively support cell attachment, growth, and tissue integration. Notably, the smallest pores are observed in fibres from the 6 w/w% solution, at 0.739 ± 0.344 μm, while pore size expands to an average of 3.279 ± 1.863 μm for the 12 w/w% solution. The relationship between changes in fibre diameter and pore size with increasing concentration is shown in Fig. 5. Concentration emerges as the primary factor influencing fibre diameter in electrospinning, often adjusted to achieve the desired fibre diameter.

Solutions with higher concentrations yield charged jets that are more resistant to bending and whipping motions. As a result, these jets travel along a longer straight trajectory towards the collector before entering a shorter instability region, ultimately resulting in the production of thicker fibres.^41,42^

### 4.4 Effect of solution concentration on the mechanical properties of the PLGA fibres

**Fig. 6:**
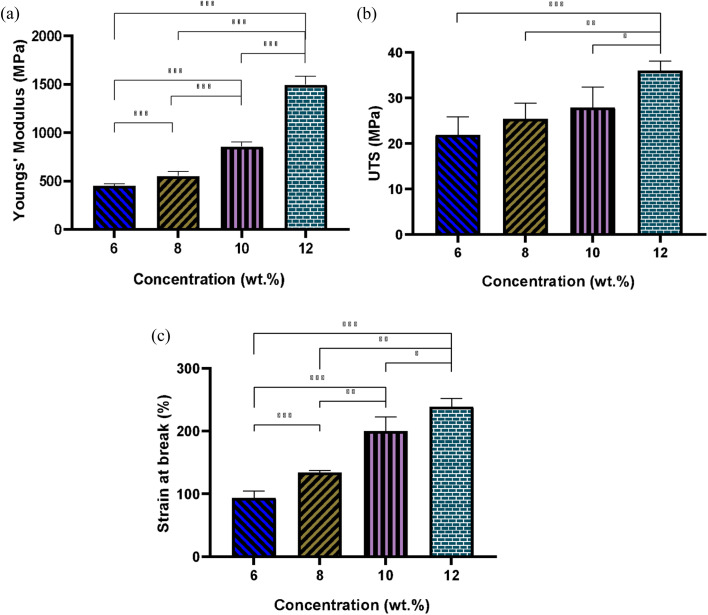
Effect of solution concentration on (a) Young's modulus, (b) UTS, and (c) strain at break of PLGA scaffolds produced from differently concentrated solutions (HFIP system); (*P < 0.05, **P < 0.001, ***P < 0.001, *N* = 5).

Fig. 6 illustrates the elastic modulus, ultimate tensile strength (UTS), and elongation at break for fibrous scaffolds fabricated from varying concentrations of PLGA solutions in HFIP. The results indicate that increasing solution concentration results in the formation of stiffer scaffolds. For instance, the Young's modulus of the scaffold produced from the 6 w/w% solution was 450.8 ± 15 MPa, whereas it nearly tripled for the 12 w/w% solution. Specifically, the elastic modulus for PG-HFIP8 and PG-HFIP10 scaffolds measured 551 ± 46 MPa and 858 ± 43 MPa, respectively. The samples also exhibited a trend of increasing UTS, with the PG-HFIP6 and PG-HFIP12 scaffolds demonstrating the weakest 21.9 ± 2 MPa and strongest 36 ± 2 MPa materials, respectively. Furthermore, the stiffest scaffolds displayed higher extension before material failure, with an increase from 93 ± 3.9 to 239 ± 13 for PG-HFIP6 and PG-HFIP12 scaffolds, respectively. These values are compared in Table 5.

**Table 5:** Mechanical properties of the PLGA scaffolds developed from different solution concentrations

Concentration (w/w%)	6	8	10	12
Young's modulus (MPa)	450.8 ± 15	551 ± 46	858 ± 43	1496 ± 85
UTS (MPa)	21.9 ± 2.5	25.4 ± 3.5	27.9 ± 4.5	36 ± 2
Breaking strain (%)	93 ± 3.9	134 ± 3.5	200 ± 23	239 ± 13

This investigation underscores the significant impact of fibre diameter on the mechanical properties of electrospun scaffolds. While previous research demonstrated this phenomenon for PCL fibres fabricated from HFIP compared to the CHL system, it should be noted that the solvent system differed in that case.^27^ The utilization of a common solvent can mitigate potential variables in the microstructure of scaffolds arising from significantly different solution properties. Consequently, this study lends support to the hypothesis that mechanical properties improve with increased fibre diameter in electrospun scaffolds.

The observed trends in mechanical properties in relation to fibre diameter can be justified by several underlying mechanisms in electrospinning processes. Firstly, the structural integrity of the electrospun scaffold is directly influenced by fibre diameter, with thicker fibres typically offering greater resistance to deformation and higher load-bearing capacity compared to thinner fibres due to their larger cross-sectional area, distributing applied forces more effectively.^27,42,43^ Additionally, fibre diameter influences pore size and density within the scaffold, with thicker fibres generally resulting in larger pores and lower pore density. This can impact mechanical properties such as tensile strength and elongation at break, as pore size and density affect the distribution of stress throughout the scaffold.^44^

Furthermore, thicker fibres tend to have fewer surface defects per unit volume compared to thinner fibres. Electrospun meshes with smaller fibre diameters often exhibit beaded surfaces.^45–48^ Surface defects, such as irregularities and beads, can act as stress concentrators and initiation points for failure. With fewer defects, thicker fibres are less prone to breaking under load.^49^

### 4.5 Optimizing the electrospinning process for PLGA scaffolds: achieving consistent average fibre diameter using different solvents

**Fig. 7:**
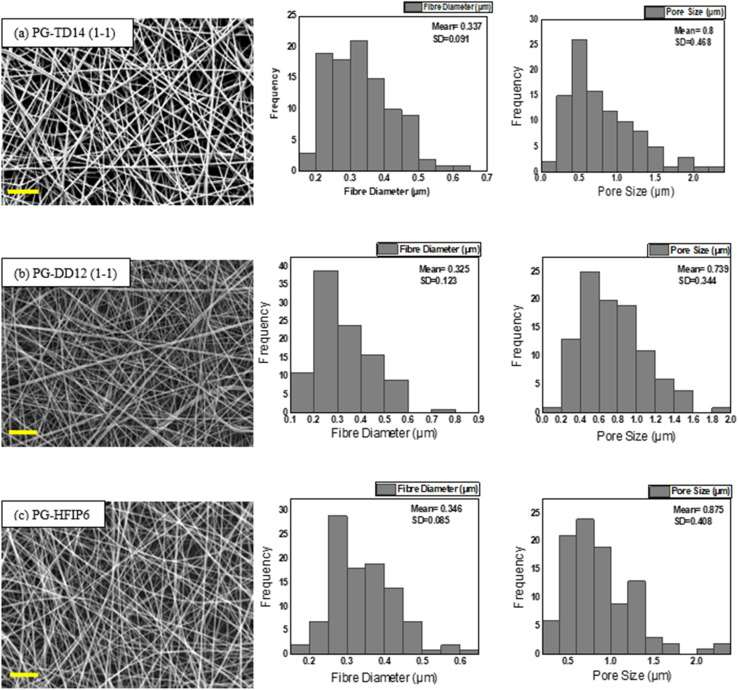
SEM images of PLGA scaffolds produced from different solvent systems and their corresponding fibre diameter and pore size; (a) 14 w/w% THF-DMF (1-1), (b) 12 w/w% DCM-DMF (1-1), (c) 6 w/w% HFIP; scale bar: 10 μm.

**Fig. 8:**
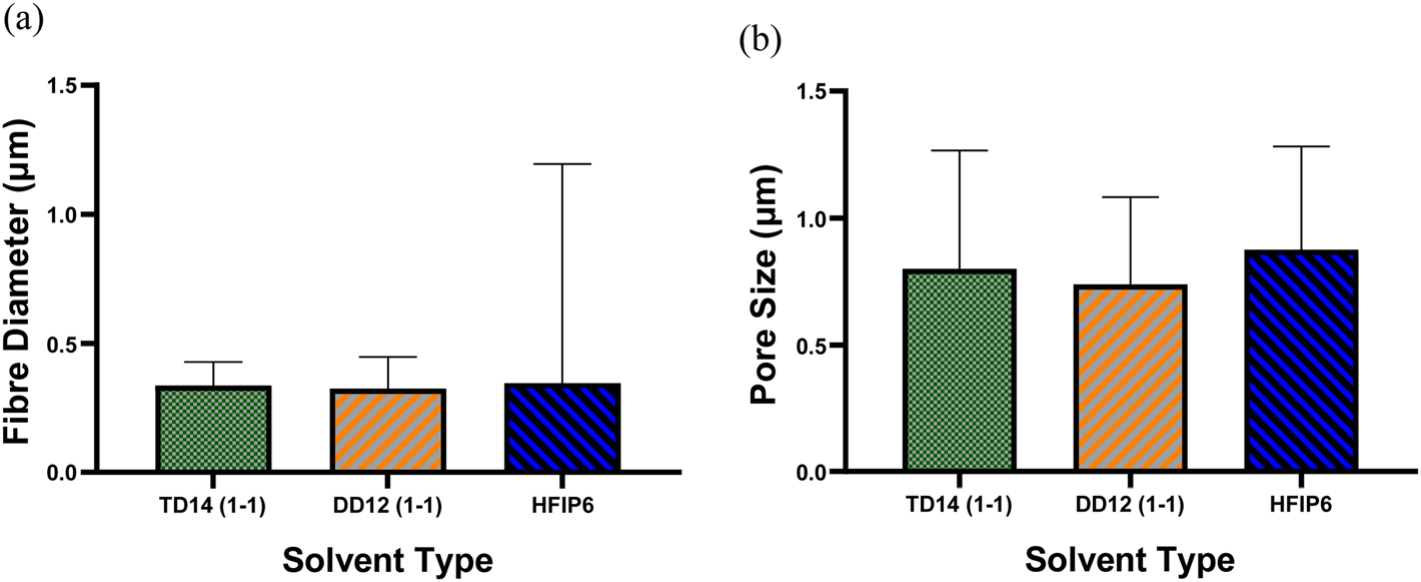
Comparison of (a) average fibre diameter and (b) pore size for scaffolds from different solvent systems; (***P < 0.001, *N* = 5).

Due to the significant impact of fibre diameter on the mechanical properties of the meshes, as evidenced in prior experiments, this study aims to optimize electrospinning parameters to achieve fibres with similar average diameters from three selected solvent systems: DCM-DMF, THF-DMF, and HFIP. By eliminating the fibre diameter variable, we can investigate the influence of solvents on the mechanical properties of the electrospun PLGA scaffolds. Solutions including TD14 (1-1), DD12 (1-1), and HFIP6 were chosen for further investigation based on the quality of fibre morphology observed in the screening trial. SEM images in Fig. 7 show fibres from the selected solvent systems, all showing comparable fibre diameters with average values of 0.337 μm, 0.325 μm, and 0.346 μm, respectively. Likewise, pore sizes were comparable, measuring 0.8 μm, 0.739 μm, and 0.875 μm for THF-DMF (1-1), DCM-DMF (1-1), and HFIP systems, respectively, as illustrated in Fig. 7. No statistically significant differences were observed in these properties among the developed scaffolds. In optimizing parameters to achieve fibres with comparable diameters, higher concentrations were used for THF and DCM systems compared to HFIP. Despite HFIP's high viscosity (1.65 cP), which is nearly one-fourth that of THF, the resulting high-viscosity solution tends to produce thicker fibres due to increased resistance against electrostatic forces, hindering polymer chain stretching during jet flight. Additionally, solution conductivity plays a crucial role, with highly conductive solutions yielding thinner fibres. While HFIP possesses a higher dielectric constant than THF and DCM, the inclusion of DMF compensates for this disparity, ensuring uniform and high-quality fibres. This phenomenon is consistent with previous findings,^50^ where higher solution conductivity resulted in thinner fibres. Additionally, lower viscosity solutions tend to produce beaded fibres due to excessive solvent molecules relative to polymer chains within the jet, whereas higher viscosity solutions promote a more homogenous distribution of solvent, leading to smoother fibre formation (Fig. 8).^51^

### 4.6 Mechanical properties of optimized PLGA scaffolds with consistent fibre diameter and pore size across various solvents

**Fig. 9:**
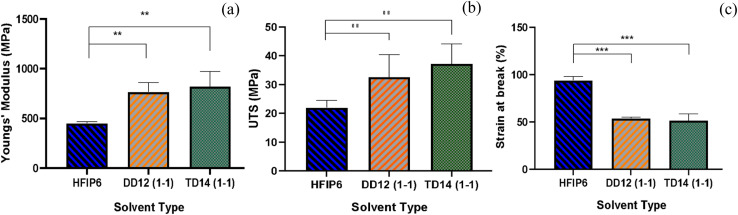
Comparisons of (a) Young's modulus, (b) UTS, and (c) strain at break of the scaffolds with comparable morphology prepared from various solvent systems; (**P < 0.001,***P < 0.001, *N* = 5).

The tensile mechanical properties of PLGA fibrous scaffolds fabricated from TD14 (1-1), DD12 (1-1), and HFIP6 solvent systems reveal distinct mechanical characteristics and behaviours under tensile loading. Fibres from the HFIP system exhibited the lowest Young's modulus, averaging 450.87 MPa, while THF-derived scaffolds were the stiffest with an average modulus of 820 MPa. The DCM system yielded scaffolds with elasticity falling in between, averaging 765.9 MPa. Ultimate stress (UTS) results varied from an average of 21.9 MPa (HFIP) to 37.17 MPa (THF), as shown in Fig. 9b. Fig. 9c illustrates the maximum elongation at break, with HFIP fibres displaying the highest strain value before failure (average of 93.9%). THF fibres exhibit an average strain value of 51.458%, and DCM fibres show an average strain value of 53.85%, with no statistically significant difference between them. Ultimate tensile stress, breaking strain, and Young's modulus for the scaffolds are detailed in Table 6.

**Table 6:** Mechanical properties of the PLGA electrospun scaffolds with comparable morphology using different solvents

	HFIP6	DD 12 (1-1)	TD 14 (1-1)
Young's modulus (MPa)	450.8 ± 15	765.9 ± 94	820 ± 150
UTS (MPa)	21.9 ± 2.5	32.55 ± 7.8	37.17 ± 6.9
Breaking strain (%)	93 ± 3.9	53.85 ± 1.1	51.45 ± 6.8

**Fig. 10:**
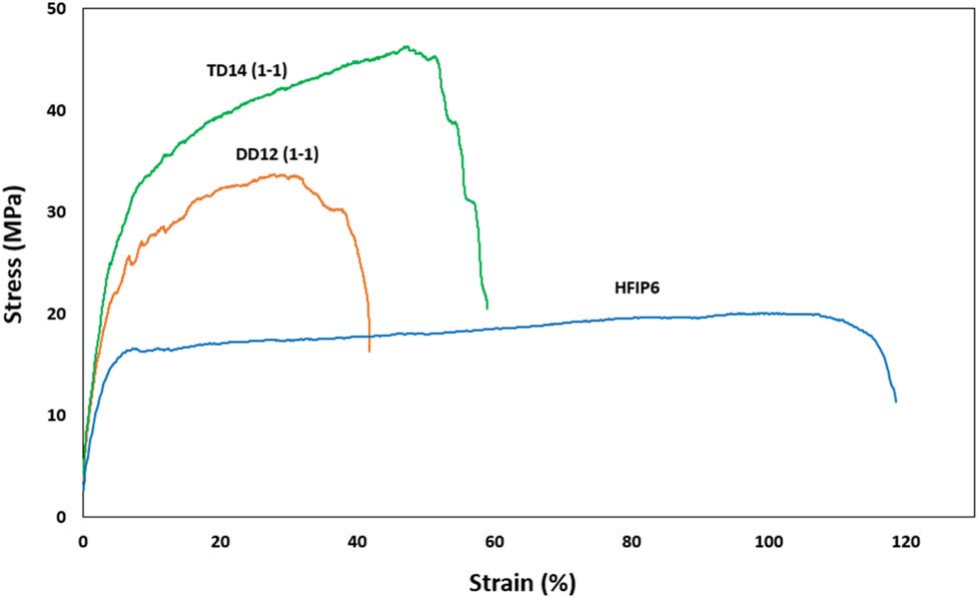
Stress–strain curves for scaffolds with comparable morphology prepared from different solvents (THF-DMF 1-1 v/v), DCM-DMF (1-1 v/v), and HFIP.

Fig. 10 depicts the stress–strain behaviour of PLGA scaffolds from the three solvent systems, highlighting differences in the mechanical response and deformation mechanisms of the PLGA scaffolds. TD14 scaffolds exhibit clear stress stiffening, where the stress increases sharply with strain, indicating strong fibre alignment and resistance to deformation. This is followed by a necking region, where the material undergoes localized plastic deformation, resulting in a pronounced peak stress before failure. DD12 scaffolds display a similar but less pronounced behaviour, with a more gradual increase in stress and a smaller necking region, suggesting a balance between fibre alignment and plastic deformation. HFIP scaffolds, on the other hand, show a distinct lack of stress stiffening and necking. The stress increases gradually and steadily, indicating a more ductile behaviour with extensive elongation before failure, fibres spun from HFIP.

The electrospinning parameters were meticulously fine-tuned to produce fibres with comparable diameters across the different solvent systems, aiming to isolate the influence of solvent choice on the mechanical properties. Despite the similar average fibre diameter, the significant differences in mechanical properties of the scaffolds suggest that various factors beyond diameter, such as fibre packing density, inter-fibre connectivity, polymer chain alignment, and crystallinity, influenced by solvent selection, play crucial roles.

The choice of solvent impacts the solubility and interaction between the polymer and solvent molecules, affecting polymer chain entanglement, molecular alignment, and intermolecular forces within the fibres. Solution viscosity is a critical factor, as it determines the flow behaviour of the polymer solution and the stretching of polymer chains during fibre formation. Higher viscosity solutions lead to a more uniform solvent distribution within the jet, resulting in smoother fibre formation with greater chain alignment and entanglement, leading to higher mechanical strength.^52^ Additionally, solution conductivity influences the electrostatic forces acting on the polymer jet during spinning; enhanced conductivity increases the forces within the jet due to greater surface charge repulsion. This elevates bending instabilities, ultimately resulting in a more elongated jet, and alignment of polymer chains, contributing to stronger and stiffer fibres.^53^ Intermolecular bonding between polymer chains within the fibres also significantly affects mechanical properties, with solvent choice impacting the degree of polymer chain entanglement and bonding interactions, ultimately influencing mechanical properties.^54^

**Fig. 11:**
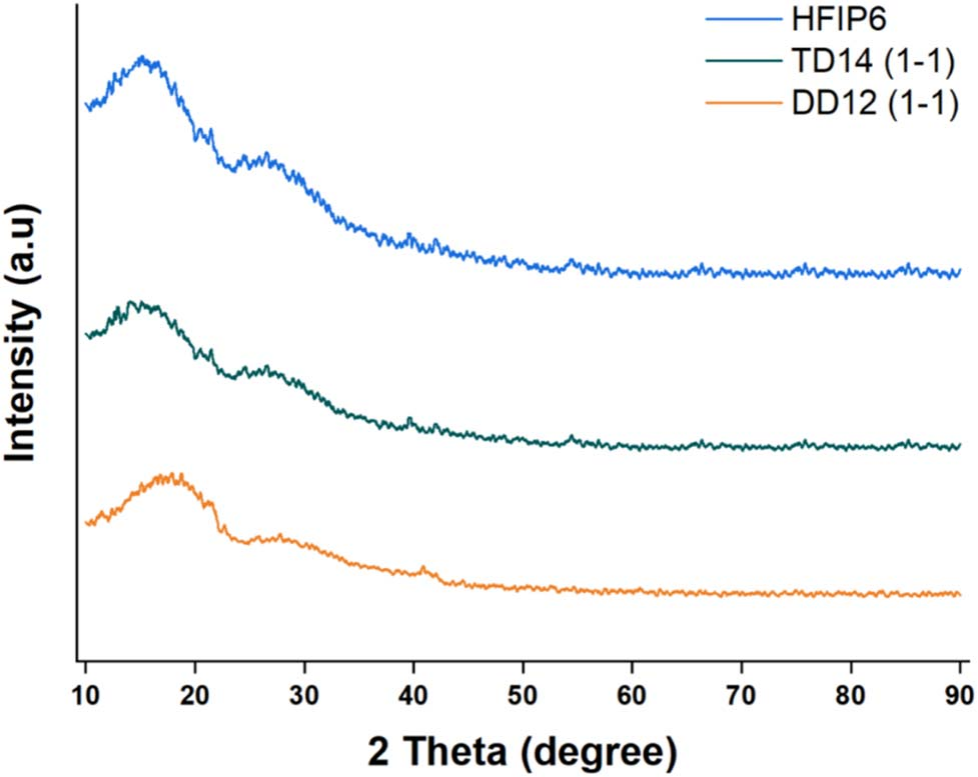
XRD profiles of PLGA fibrous scaffolds made from solvent systems including DCM-DMF (1-1, v/v), THF-DMF DCM-DMF (1-1, v/v), and HFIP.

Electrospun scaffolds prepared from PLGA using various solvent systems, including DCM–DMF (1 : 1 v/v), THF-DMF, and HFIP, were analysed for crystallinity using X-ray diffraction (XRD). The results, presented in Fig. 11, indicate that none of the scaffolds exhibited distinct crystallinity peaks. The absence of such peaks is a clear indicator that no significant crystallite formation occurred during the electrospinning process, thus confirming the amorphous nature of these PLGA scaffolds, regardless of the solvent system used.

This lack of crystallinity is not unusual for PLGA, as the random copolymerization of lactic acid and glycolic acid disrupts the regular arrangement of polymer chains, making it difficult for crystallites to form. Given that all the scaffolds shared this amorphous character, the differences in mechanical properties observed between the scaffolds are unlikely to stem from crystallinity variations.

**Fig. 12:**
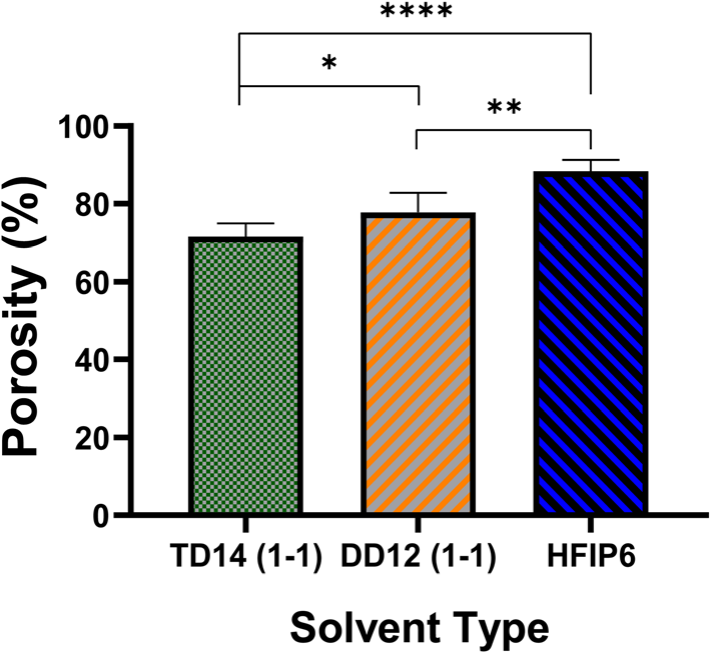
The porosity of electrospun PLGA scaffolds prepared from various solvent systems; (*P < 0.05, **P < 0.001, ***P < 0.001, *N* = 5).

Porosity analysis, on the other hand, as presented in Fig. 12, highlights variations among the scaffolds, with HFIP6 exhibiting the highest porosity and TD14 displaying the lowest. This disparity can be attributed to the differing volatilities of the solvents utilized. DCM, the most volatile solvent in the selection, combined with DMF (DCM-DMF: 1-1), a relatively less volatile organic solvent with a boiling point of 153 °C, still exhibited greater volatility compared to THF-DMF: 1-1, where THF has a boiling point of 66 °C. HFIP, with a boiling point of 59 °C, stands out as the most volatile solvent investigated herein. This volatility disparity likely contributes to the observed variations in scaffold porosity, which in turn may influence their mechanical properties.

HFIP's higher porosity suggests lower fibre packing density and weaker inter-fibre bonding, leading to lower tensile strength but higher extensibility. In contrast, TD14's lower porosity indicates denser fibre packing and stronger inter-fibre bonding, resulting in higher tensile strength and moderate extensibility.

Solvent choice may also influence the degradation behaviour of the PLGA scaffolds, and that in turn could affect the mechanical properties. The hydrolysis of PLGA's ester linkages, which underpins its degradation process, can be affected by scaffold porosity. For instance, the solvent system used may alter the porosity and fibre morphology, which in turn impacts water absorption and hydrolysis rates.

**Fig. 13:**
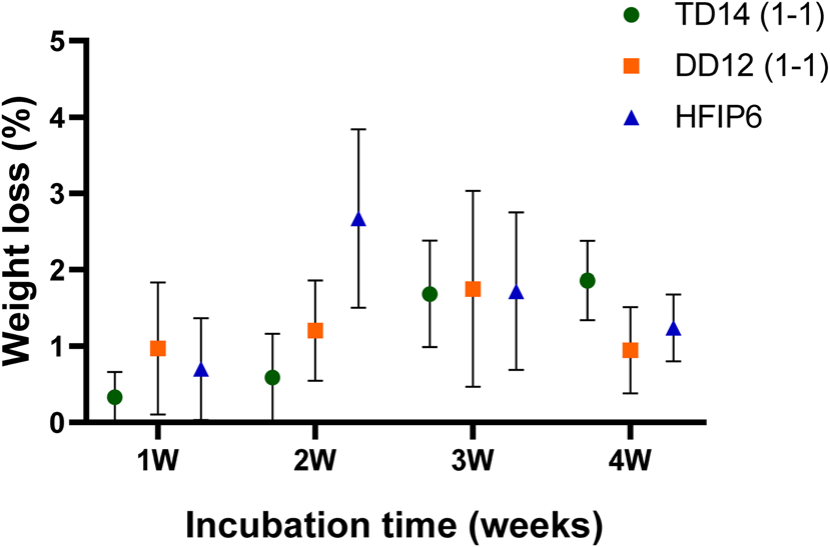
Degradation profiles of electrospun PLGA scaffolds prepared from various solvent systems over a period of 4 weeks.

Although no significant weight loss was observed during the one-month degradation study we conducted under simulated biological conditions (37 °C in PBS), nor any statistically significant changes for scaffolds made using different solvents (see Fig. 13), this relatively short timeframe may not fully capture the long-term degradability of the scaffolds, particularly under application-specific loading conditions. Further investigation is necessary to explore how different solvent systems affect the degradation profile, particularly in relation to changes in mechanical integrity over time. This is critical because the gradual loss of mechanical properties, due to polymer degradation, could significantly influence the scaffold's ability to maintain structural support or elasticity during tissue regeneration.

Understanding the mechanical properties of PLGA scaffolds is critical for tailoring these scaffolds for specific biomedical applications. TD14 scaffolds, with their high tensile strength and stress stiffening, are suitable for applications requiring robust structural support, such as load-bearing tissue engineering scaffolds, *e.g.* bone regeneration. DD12 scaffolds offer a balanced profile with moderate stress stiffening, making them versatile for general tissue engineering applications where both strength and flexibility are desired, *e.g.* meniscus and tendon regeneration. HFIP scaffolds, characterized by their high extensibility and ductile behaviour without significant stress stiffening, are ideal for applications requiring significant flexibility, such as soft tissue engineering or wound healing. This understanding allows for the strategic optimization of electrospun PLGA scaffolds to meet specific biomedical application requirements. Understanding the influence of solvent selection on these factors allows for the strategic optimization of electrospun PLGA scaffolds, tailoring them to meet specific biomedical application requirements.

## 5. Conclusion

This work provides new empirical determination of the Hansen solubility parameters for PLGA and a systematic investigation of various solvent systems' impact on the microstructure of electrospun scaffolds. The establishment of PLGA solubility parameters through Hansen solubility theory marks a significant contribution, providing a systematic foundation for solvent selection in scaffold fabrication. Achieving consistent fibre diameter and isolating the solvent's influence on mechanical properties advances the understanding and optimization of electrospun PLGA scaffolds for biomedical applications. Suitable solvent systems, including THF, DCM, and HFIP, were identified, crucial for achieving uniform fibre morphology and enhancing mechanical characteristics. Morphological analysis revealed the importance of solvent concentration and combinations in minimizing bead formation and ensuring defect-free fibres. Mechanical characterisation demonstrated significant variations in stiffness and elasticity across different solvent systems, emphasizing the critical role of solvent selection in scaffold fabrication. Overall, this research offers valuable insights into tailoring solvent systems for improved scaffold functionality in tissue engineering, fostering advancements in biomedical research and applications.

## Data availability

The authors declare that the data supporting the findings of this study are available within the paper. Should any raw data files be needed in another format they are available from the corresponding author upon reasonable request.

## Conflicts of interest

There are no conflicts to declare.
